# Detection of pre-existing neutralizing antibodies against Ad26 in HIV-1-infected individuals not responding to the Ad26.COV2.S vaccine

**DOI:** 10.1007/s15010-023-02035-6

**Published:** 2023-04-17

**Authors:** Katja G. Schmidt, Ellen G. Harrer, Verena Schönau, David Simon, Arnd Kleyer, Philipp Steininger, Klaus Korn, Georg Schett, Carina S. Knobloch, Krystelle Nganou-Makamdop, Thomas Harrer

**Affiliations:** 1 Infectious Diseases and Immunodeficiency Section, Department of Internal Medicine 3, Universitätsklinikum Erlangen, Friedrich-Alexander-Universität Erlangen-Nürnberg, Erlangen, Germany; 2 Department of Internal Medicine 3, Rheumatology and Immunology, Universitätsklinikum Erlangen, Friedrich-Alexander-Universität Erlangen-Nürnberg, Erlangen, Germany; 3 Institute of Clinical and Molecular Virology, Universitätsklinikum Erlangen, Friedrich-Alexander-Universität Erlangen-Nürnberg, Erlangen, Germany; 4 Institute and Outpatient Clinic of Occupational, Social and Environmental Medicine, Friedrich-Alexander-Universität Erlangen-Nürnberg, Erlangen, Germany; 5 Vaccination Center Erlangen, Erlangen, Germany

**Keywords:** SARS-CoV-2, HIV-1, Ad26.COV2.S, SARS-CoV-2 vaccine, Adenovirus 26, Neutralizing antibodies

## Abstract

**Purpose:**

The Ad26.COV2.S vaccine is a replication-incompetent human adenovirus type 26 vector encoding the SARS-CoV-2 spike protein. In a phase 1-2a trial, a single dose of Ad26.COV2.S induced SARS-CoV-2 spike-specific antibodies in ≥ 96% of healthy adults. To investigate vaccine immunogenicity in HIV-1-infection, we measured SARS-CoV-2 spike-specific antibodies in Ad26.COV2.S vaccinated HIV-1-infected patients and analyzed the presence of pre-existing Ad26 neutralizing antibodies.

**Methods:**

We included all Ad26.COV2.S vaccinated HIV-1-infected patients of Erlangen HIV cohort fulfilling all inclusion criteria. The study cohort consisted of 15 HIV-1-infected patients and three HIV-1-uninfected subjects who received the Ad26.COV2.S vaccine between April and November 2021. Pre-vaccination sera were collected between October 2014 and June 2021, post-vaccination sera between June and December 2021. Neutralizing antibodies towards Ad26 were determined by a FACS-based inhibition assay measuring the expression of SARS-CoV-2 spike and adenoviral proteins in HEK293T cells after in-vitro transduction with Ad26.COV2.S or the control ChAdOx1-S.

**Results:**

Six out of 15 HIV-1-infected patients failed to develop SARS-CoV-2-specific antibodies and four patients developed weak antibody responses after vaccination with Ad26.COV2.S. Pre-vaccination sera of four of the six vaccine non-responders showed neutralizing activity towards Ad26.COV2.S but not toward the ChAdOx1-S vaccine at 1:50 dilution. After Ad26.COV2.S vaccination, 17 of the 18 subjects developed strong Ad26-neutralizing activity and only one of the 18 subjects showed neutralizing activity towards the ChAdOx1-S vaccine.

**Conclusion:**

Ad26.COV2.S vaccination showed a high failure rate in HIV-1-infected patients. Pre-existing immunity against Ad26 could be an important contributor to poor vaccine efficacy in a subgroup of patients.

**Supplementary Information:**

The online version contains supplementary material available at 10.1007/s15010-023-02035-6.

## Introduction

Vaccination against the severe acute respiratory syndrome coronavirus 2 (SARS-CoV-2) has evolved as an effective strategy to prevent a severe course of COVID-19 [1–3], even in immunodeficient patient groups such as HIV-1-infected patients [4, 5]. It has been shown by several groups that vaccination with BNT162b2 mRNA, mRNA-1273 and ChAdOx1-S was able to induce SARS-CoV-2-spike specific IgG antibodies in HIV-1-infected patients comparable to healthy subjects [6–9]. In a recent study of our group, all 50 enrolled HIV-1-infected subjects on antiretroviral therapy developed SARS-CoV-2-specific IgG antibodies after two doses of the BNT162b2 mRNA vaccine [9].

While there is a growing experience in HIV-1 infected persons with the use of several vaccines such as the two approved mRNA COVID-19 vaccines [5], the ChAdOx1-S vaccine [6], the NVX-CoV2373 vaccine (Novavax) [10], the Sputnik vaccine [11], and the inactivated COVID-19 vaccine WIBP-CorV (Sinopharm) [12], less data are available regarding the immunogenicity of the Ad26.COV2.S vaccine (Jcovden, previously known as COVID-19 Vaccine Janssen) in HIV-1-infected patients. The Ad26.COV2.S vaccine is a replication-incompetent human adenovirus type 26 vector encoding the SARS-CoV-2 spike protein. As the Ad26.COV2.S vaccine has been licensed later than the mRNA-based vaccines and the ChAdOx1-S vaccine, it has been used much less frequently in Germany with a proportion of only 2.03% of all COVID-19 vaccinations (vaccine monitoring of the Robert Koch Institute, online, 28^th^ of January 2023: https://impfdashboard.de/daten). In the phase 1-2a trial, a single dose of Ad26.COV2.S induced SARS-CoV-2-spike specific antibodies in > 96% of the vaccinees in the various patient cohorts [3]. In the phase 3 Ensemble trial, a single administration of the Ad26.COV2.S vaccine demonstrated a vaccine efficacy of 56.3% against moderate to severe COVID-19 [13]. Subgroup analysis of the Ensemble trial indicated a lower vaccine efficacy against moderate to severe COVID-19 in HIV-1-infected participants (15.5%) in comparison to HIV-1-uninfected vaccinees (56.9%).

To assess the vaccine efficacy of the Ad26.COV2.S vaccine in HIV-1-infected patients, we retrospectively analyzed the immunogenicity of the Ad26.COV2.S vaccine in HIV-1-infected subjects of the Erlangen HIV cohort. As we observed a high failure rate to develop SARS-CoV-2 specific antibodies, we investigated pre-existing anti-vector immunity as a potential cause for poor vaccine response towards Ad26.COV2.S. For this purpose, we measured neutralizing antibodies (nAbs) against the Ad26.COV2.S vaccine in serum samples collected before and after vaccination with Ad26.COV2.S.

## Material and methods

### Study subjects

We included all 15 HIV-1-infected patients from the Erlangen HIV cohort who had been vaccinated once with Ad26.COV.2 and who met all inclusion criteria and none of the exclusion criteria: Inclusion criteria were HIV-1-infection on combination antiretroviral therapy (cART) at the time of vaccination, a CD4 count of > 200/µl at the evaluation before and after vaccination with Ad26.COV2.S and availability of a pre-vaccination serum or plasma sample. Exclusion criteria were an overt disease, HIV-unrelated immunodeficiency, prior Covid-19 and vaccination with another SARS-CoV-2 vaccine before the post-vaccination evaluation. None of the patients had hepatitis C co-infection. All subjects grew up in Central European or Eastern European countries except for one subject (#15) who immigrated from Brazil. Characteristics of the study participants are summarized in supplementary Table S1. At the post-vaccination evaluation time point, the median CD4 count was 702/µl (range 281–1177/µl) and the median viral load was < 20 copies/ml (range < 20–26 000 copies/ml). Except for one ART-non-compliant patient (#15) who presented with a high viral load of 26 000 copies/ml, all other patients showed an undetectable or a low viral load of ≤ 50 copies/ml at the post-vaccination evaluation time point. In addition, we screened for Ad26.COV.2 vaccinated subjects in a cohort of healthy subjects who participated in a prospective SARS-CoV-2 seroprevalence study [14]. We identified three subjects with available pre-vaccination blood sample who could be included in this analysis. Two of these HIV-1-uninfected subjects had prior PCR-proven SARS-CoV-2 infection with detectable antibody titers at the time of pre-vaccination serum collection. All analyzed subjects had been vaccinated externally in COVID-19 vaccine centers or by their private practitioners between May and November 2021.

SARS-CoV-2 antibody titers were measured in the HIV-outpatient clinic of the Department of Medicine 3 at their first regular visit after vaccination. Serum (*n* = 33) or plasma (if no serum was available, *n* = 3) samples were collected for the analysis of pathogen- or vaccine-specific immune responses and were stored at  – 20 °C until use. All samples will be referred to as sera in the manuscript. Four sera were from pre-pandemic time points, the other sera were collected between May 2020 and November 2021. The Erlangen HIV cohort is comprising all HIV-1-infected patients treated at the outpatient ward of the Department of Medicine 3 of the University Hospital Erlangen. The study with analysis of virus-specific immune responses was approved by the Ethics Committee of the Medical Faculty (Number 250_15B and 157_20B). Blood was obtained after written informed consent.

### Measurement of SARS-CoV-2 IgG antibodies

IgG antibodies against the SARS-CoV-2 spike protein were detected using two commercial assays (LIAISON SARS-CoV-2 TrimericS IgG assay, Diasorin, Dietzenbach, Germany; SARS-CoV-2 IgG ELISA, Euroimmun, Lübeck, Germany) [15, 16]. Values of ≥ 33.8 Binding antibody units (BAU)/ml (LIAISON) or a ratio of ≥ 1.1 (Euroimmun) were considered positive according to the instruction of the manufacturer. A ratio of 0.8 to 1.1 in the Euroimmun assay was considered borderline.

### *In-vitro* transduction of HEK293T with Ad26.COV2.S or ChAdOx1-S vaccine

HEK293T cells (provided by M. Tenbusch, Institute of Virology, Erlangen, Germany) were seeded in a 48-well plate (ThermoFisher Scientific, Waltham, Massachusetts, USA) in D10 medium (Dulbecco’s Modified Eagle Medium (DMEM), ThermoFisher Scientific, 10% heat-inactivated fetal bovine serum (FCS, PAN Biotech GmbH, Aidenbach, Germany), 1% L-glutamine (2 mmol/l, ThermoFisher Scientific), penicillin (100 U/ml, ThermoFisher Scientific), streptomycin (100 μg/ml, ThermoFisher Scientific), Hepes (10 mmol/l, Merck KGaA, Darmstadt, Germany)) at 1 × 10^5^ cells/well. The next day, half of the medium was replaced with a D10 medium containing the Ad26.COV2.S vaccine (Lot number: XE395, Johnson & Johnson, New Brunswick, New Jersey, USA) or the ChAdOx1-S vaccine (Lot number: ABW2586, AstraZeneca, Cambridge, UK) and/or the respective sera. The vaccines were pre-incubated with the sera for 15 min at room temperature prior to adding them to the cells. The cells were transduced with the vaccines at a final concentration of 2.5 × 10^8^ viral particles/well (≙1 × 10^9^ viral particles/ml). Serum was added at a final dilution of 1:200, 1:100 and 1:50, respectively. The vaccines were provided by the Vaccination Center Erlangen.

### Flow-cytometric analysis of spike- and adenovirus-expression

24 h after transduction, cells were harvested and washed once with PBS at 400xg for 5 min at room temperature. Viability was assessed by staining with Zombie Aqua (BioLegend, San Diego, California, USA) at a final dilution of 1:1000 for 20 min at room temperature followed by washing with FACS buffer (PBS (ThermoFisher Scientific), 0.5% BSA (Carl Roth, Karlsruhe, Germany), 2 mM EDTA (Merck KGaA)). Following a 15 min fixation with 4% PFA (Merck KGaA) at 4 °C, cells were washed with 1 ml FACS buffer. After permeabilization with 200 µl perm buffer (PBS (ThermoFisher Scientific), 1% BSA (Carl Roth), 0.3% Saponin (Merck Millipore, Burlington, Massachusetts, USA)) for 20 min at room temperature, cells were centrifuged and stained with rat α-spike antibody (final dilution 1:100, Clone A20103O, BioLegend) and mouse α-adeno antibody (final dilution 1:5, Merck Millipore) in perm buffer for 15 min at room temperature. The cells were washed with 1 ml perm buffer and then stained for 15 min at room temperature with goat α-rat APC (final dilution 1:2000, Clone Poly4054, BioLegend) and goat α-mouse FITC (final dilution 1:1000, Southern Biotech, Birmingham, Alabama, USA) in perm buffer. After washing with 1 ml FACS buffer, cells were resuspended in 300 µl MACS buffer and measured on a Navios flow cytometer (Beckman-Coulter, Brea, California, USA). Analysis was carried out using FlowJo v10.8.0 (BD Biosciences, Franklin Lakes, New Jersey, USA) and GraphPad Prism 9.0. (GraphPad Software Inc., San Diego, California, USA). The gating strategy and an example of the flow cytometric analyses are shown in supplementary Fig. S1. To compare the relative reduction of adenoviral and spike protein expression, their expression after the addition of sera/plasma was normalized to the expression level in absence of serum/plasma.

### Statistical analysis

Mann–Whitney-*U* test was used for comparison of neutralizing activity, antibody titers, CD4 T cell counts, viral loads, age and timepoints between groups. Wilcoxon signed-rank test was applied for comparison of antibody titers before and after booster vaccination as well as CD4 counts and viral loads before and after vaccination. All statistical analysis was carried out using GraphPad Prism 9.0.2 (GraphPad Software Inc.).

## Results

### Poor immunogenicity of the Ad26.COV2.S vaccine in HIV-1-infected subjects of the Erlangen HIV-1 cohort

To investigate the immunogenicity of the Ad26.COV2.S vaccine in HIV-1-infected subjects, we performed a retrospective analysis of SARS-CoV-2-spike specific IgG antibodies in all 15 HIV-1-infected subjects of the Erlangen HIV cohort who had been vaccinated once with Ad26.COV.2 and who met all inclusion criteria and none of the exclusion criteria. All were on antiretroviral therapy with a CD4 T cell count > 200/µl. Six of the 15 (40%) HIV-1-infected subjects were non-responders without a spike-specific IgG response after vaccination with Ad26.COV2.S (< 33.8 BAU/ml) and four patients (26.7%) were low-responders exhibiting only low antibody titers after Ad26.COV2.S vaccination (33.8–100 BAU/ml). Only 5 subjects (33.3%) developed spike-specific IgG levels of > 100 BAU/ml after vaccination with Ad26.COV2.S (classified as responders, Table 1).

**Table 1:** SARS-CoV-2-specific antibody titers after vaccination

ID	Age^a^	Sex	Responder status	Days post Vaccination^b^	Antibody titer^c^	Antibody titer^d^
# 1	39	M	Responder	21d	9.2	1890
# 2	52	M	Responder	110d	5.3	329
# 3	38	F	Responder	72d	4.3	318
# 4	41	M	Responder	75d	3.2	148
# 5	45	F	Responder	72d	7.1	519
Median^e^	41 [39–49]			72 [47–93]	5.3 [3.5–8.2]	329 [233–1205]
# 6	28	M	Low responder	101d	0.6	51.5
# 7	54	M	Low responder	64d	1.0	93.1
# 8	43	M	Low responder	26d	0.9	73.7
# 9	64	M	Low responder	69d	2.0	76.9
Median	49 [32–62]			67 [36–93]	1.0 [0.6–1.8]	75 [57–89]
# 10	64	M	Non-responder	64d	0.9	<4.81
# 11	43	M	Non-responder	21d	0.2^f^	<4.81
# 12	55	M	Non-responder	109d	0.3	<4.81
# 13	71	M	Non-responder	117d	0.8	12.5
# 14	35	M	Non-responder	11d	0.1^ g^	8.42
# 15	46	F	Non-responder	129d	0.6	10.4
Median	51 [41–66]			87 [19–120]	0.5 [0.2–0.8]	6.6 [4.8–11]

^a^Age at the time of blood sampling after vaccination

^b^Time between blood sampling of post-vaccination serum and vaccination

^c^SARS-CoV-2 IgG-antibody titer after vaccination as determined by ELISA (Euroimmun). The cutoff for a positive result was at 1.1

^d^SARS-CoV-2 IgG-Antibody titer after vaccination as determined by LIAISON® SARS-CoV-2 TrimericS IgG (Diasorin). The cutoff for a positive result was 33.8 BAU/ml

^e^Medians with interquartile ranges (IQRs) in brackets

^f^111d after vaccination, the antibody titer of NR2 was still negative with a ratio of 0.3

^g^60d after vaccination, the antibody titer of #14 was still negative with a ratio of 0.7. Subjects were classified as responders, low responders, and non-responders according to the results of the LIASON assay as this assay indicates the results in BAU/ml according to the WHO standard

Overall, antibody responses as measured by the LIAISON assay in our cohort of 18 individuals were confirmed by the Euroimmun assay with significantly higher median titers in the responders than in the low responders and the non-responders. There were no significant differences regarding CD4 counts, viral loads, age, and length of antiretroviral therapy between the responders and the groups of low responders and non-responders (supplementary Table S1). As we had observed a good vaccine response to BNT162b2 mRNA vaccination in a similar group of HIV-1-infected subjects from the Erlangen HIV cohort, we assumed that the low vaccine response rate to Ad26.COV2.S was unlikely caused by HIV-1-induced immunodeficiency alone. HIV-1-unrelated immunoglobulin G deficiency could be ruled out as all HIV-1-infected patients had normal or even elevated serum gammaglobulins at both evaluation time points before and after vaccination as determined by standard serum electrophoresis (supplementary Table S1).

### Ad26.COV2.S vaccine non-responders show neutralizing activity towards Ad26 in pre-vaccination sera

To determine whether pre-existing Ad26-specific antibodies could have contributed to low vaccine immunogenicity in our HIV-1 cohort, we measured the neutralizing activity towards Ad26 in pre- and post-vaccination serum or plasma samples in the 15 HIV-1-infected subjects. In addition, we analyzed three healthy HIV-1-uninfected volunteers with available pre-vaccination serum samples who had developed spike-specific IgG after Ad26.COV2.S vaccination (two vaccine responders (#16 and #17) and one low responder (#18)). The two HIV-1-uninfected vaccine responders (#16 and #17) had prior PCR-proven SARS-CoV-2 infection with detectable antibody titers at the time of pre-vaccination serum collection (supplementary Table S1). None of the other donors had SARS-CoV-2 specific antibodies in their pre-vaccination sera.

For the assessment of pre-existing nAbs against Ad26, the capacity of pre-vaccination sera to block vaccine-induced spike and adenoviral protein expression in HEK293T cells was tested. The ChAdOx1-S vaccine, which is based on a chimpanzee adenovirus served as a control.

**Fig. 1:**
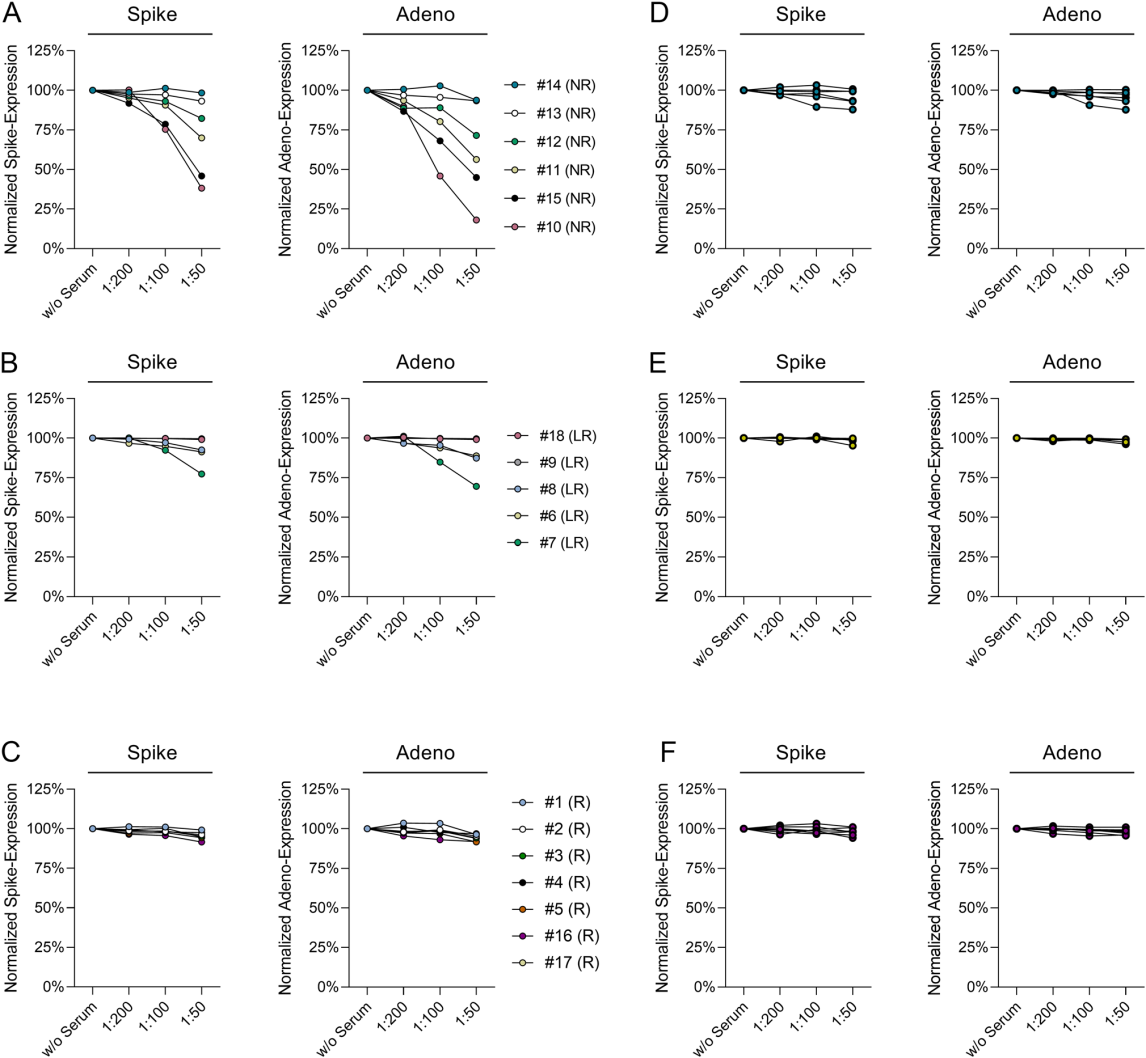
Blocking of vaccine-induced spike and adenoviral protein expression in HEK293T cells after addition of pre-vaccination sera. **A** Expression of spike and adenoviral protein induced by Ad26.COV2.S after the addition of pre-vaccination sera of the 6 vaccine non-responders. **B** Expression of spike and adenoviral protein induced by Ad26.COV2.S after the addition of pre-vaccination sera of the 5 vaccine low responders. **C** Expression of spike and adenoviral protein induced by Ad26.COV2.S after the addition of pre-vaccination sera of the 7 vaccine responders. **D** Expression of spike and adenoviral protein induced by ChAdOx1-S after addition of pre-vaccination sera of the 6 vaccine non-responders. **E** Expression of spike and adenoviral protein induced by ChAdOx1-S after addition of pre-vaccination sera of the 5 vaccine low responders. **F** Expression of spike and adenoviral protein induced by ChAdOx1-S after addition of pre-vaccination sera of the 7 vaccine responders

Both vaccines induced a high spike and adenoviral protein expression rate of 91–98% and 81–99%, respectively. We found that pre-vaccination sera of four out of six (66.7%) vaccine non-responders showed neutralization capacity against the Ad26.COV2.S vaccine. At a 1:50 serum dilution, the normalized spike expression decreased to about 38%, 46%, 70% and 82%, respectively (Fig. 1a, supplementary Fig. S2c, Table 2). The effect on adenoviral protein expression was even more pronounced with the reduction of normalized expression to about 18%, 45%, 56% and 72% at the 1:50 dilution, respectively. In contrast, in the group of vaccine responders the normalized spike and adenoviral protein expression stayed at high levels of at least 92% and 87%, respectively (Fig. 1c, supplementary Fig. S2a, Table 2).

**Table 2:** Normalized Spike and Adenoviral Protein Expression

	Pre-vaccination serum 1:50				Post-vaccination serum 1:50			
	Ad26.COV2.S		ChAdOx1.S		Ad26.COV2.S		ChAdOx1.S	
	Spike	Adeno	Spike	Adeno	Spike	Adeno	Spike	Adeno
#1 (R)	99%	96%	98%	96%	24%	10%	98%	98%
#2 (R)	96%	95%	98%	97%	96%	94%	99%	99%
#3 (R)	95%	94%	101%	99%	36%	29%	97%	98%
#4 (R)	98%	97%	98%	96%	8%	5%	94%	95%
#5 (R)	94%	92%	96%	99%	16%	10%	95%	98%
#16 (R)	92%	92%	101%	101%	43%	26%	94%	98%
#17 (R)	95%	94%	94%	98%	74%	58%	91%	96%
Median^a^	95 [94–98]	94 [92–96]	98 [96–101]	98 [96–99]	36 [20–59]	26 [10–43]	95 [94–96]	98 [97–98]
#6 (LR)	91%	89%	95%	96%	9%	7%	94%	95%
#7 (LR)	77%	70%	99%	99%	4%	4%	20%	24%
#8 (LR)	93%	87%	99%	99%	10%	11%	77%	89%
#9 (LR)	100%	100%	100%	100%	46%	27%	100%	98%
#18 (LR)	99%	99%	99%	98%	38%	22%	98%	96%
Median	93 [84–99]	89 [79–99]	99 [97–100]	99 [97–99]	10 [9–10]	11 [7–11]	94 [77–94]	95 [89–95]
#10 (NR)	38%	18%	99%	99%	27%	13%	100%	99%
#11 (NR)	70%	56%	88%	88%	28%	17%	94%	98%
#12 (NR)	82%	72%	93%	93%	13%	7%	93%	93%
#13 (NR)	93%	93%	101%	100%	18%	14%	98%	97%
#14 (NR)	98%	94%	99%	97%	35%	18%	94%	84%
#15 (NR)	46%	45%	93%	95%	20%	14%	94%	97%
Median	76 [44–95]	64 [38–94]	96 [92–100]	96 [92–99]	23 [19–27]	14 [13–17]	94 [94–94]	97 [94–97]

#1-#15 HIV-1-infected subjects, #16-#18: HIV-1-uninfected subjects. Plasma was used for pre-vaccination samples from patients #3, #8 and #15. All other samples were sera. ^a^Medians with IQRs in brackets

**Fig. 2:**
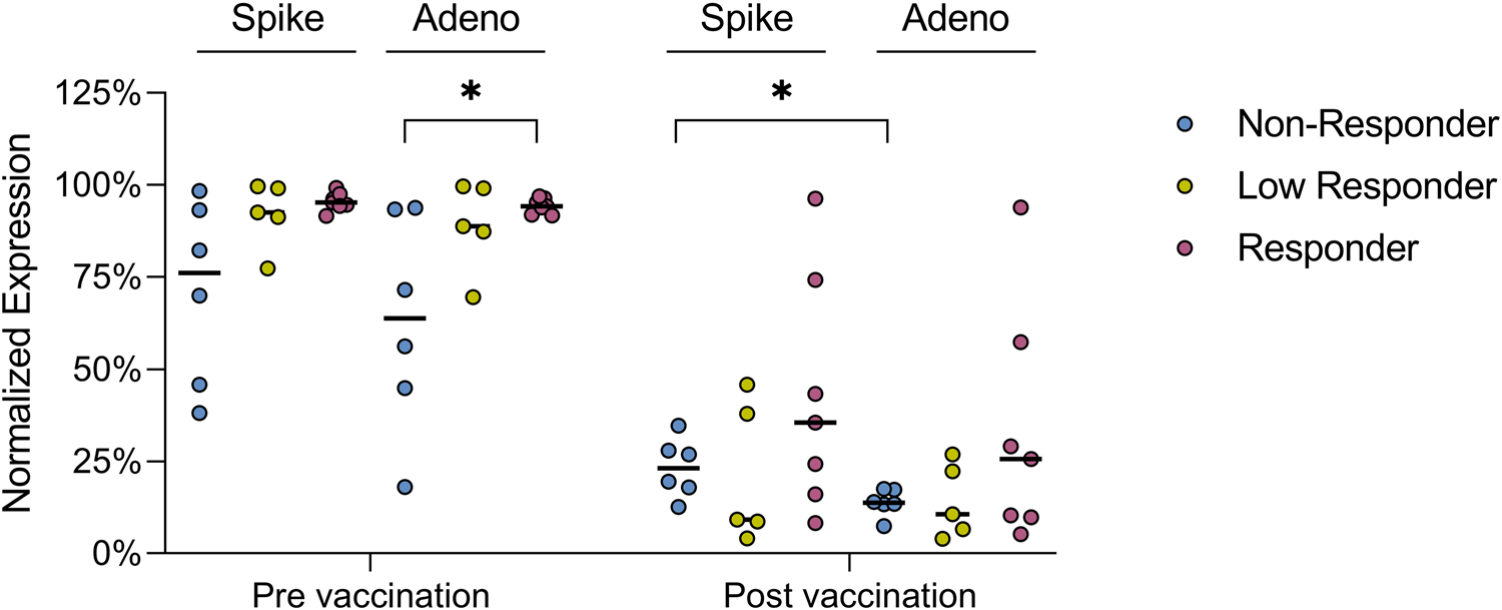
Blocking of Ad26.COV2.S-induced spike and adenoviral protein expression by pre- and post-vaccination sera. Ad26.COV2.S-induced spike and adenoviral protein expression in HEK293T cells were analyzed after the addition of pre- and post-vaccination sera in a 1:50 dilution. Significance was tested by Mann–Whitney *U* tests. * = *p* ≤ 0.05

The pre-vaccination serum of one vaccine low responder was able to reduce the Ad26.COV2.S-induced spike and adenoviral normalized protein expression to about 77% and 70%, respectively (Fig. 1b, supplementary Fig. S2b). At the 1:50 dilution, pre-vaccination sera of vaccine non-responders showed significantly higher inhibition of adenoviral protein expression compared to the group of vaccine responders (median normalized expression non-responders: 64%, responders: 94%, *p* = 0.014, Fig. 2). After vaccination, sera from all subjects except for one (#2) were capable of decreasing Ad26.COV2S transduction with reduction of spike expression to at least 74% (median 24%) and of adenoviral protein expression to at least 58% (median 14%) (Fig. 2; Table 2, supplementary Fig. S2). Both vaccine non-responders without Ad26-specific nAbs in pre-vaccination sera developed nAbs against Ad26 after vaccination.

### Analysis of serum-mediated neutralization of ChAdOx1-S

The reported seroprevalence of ChAdOx1 antibodies is lower than for Ad26 [17]. In line with this, no subject displayed neutralizing capacity towards the ChAdOx1-S vaccine in pre-vaccination sera (Fig. 1d, e and f, supplementary Fig. S3). After vaccination, nAbs towards ChAdOx1-S could be detected in the post-vaccination serum in only one of the 18 Ad26.COV2.S vaccinated subjects (#7, Table 2, supplementary Fig. S3).

### Ad26.COV2.S non-responders are able to generate SARS-CoV-2 spike-specific IgG after mRNA or protein subunit booster vaccination

**Fig. 3:**
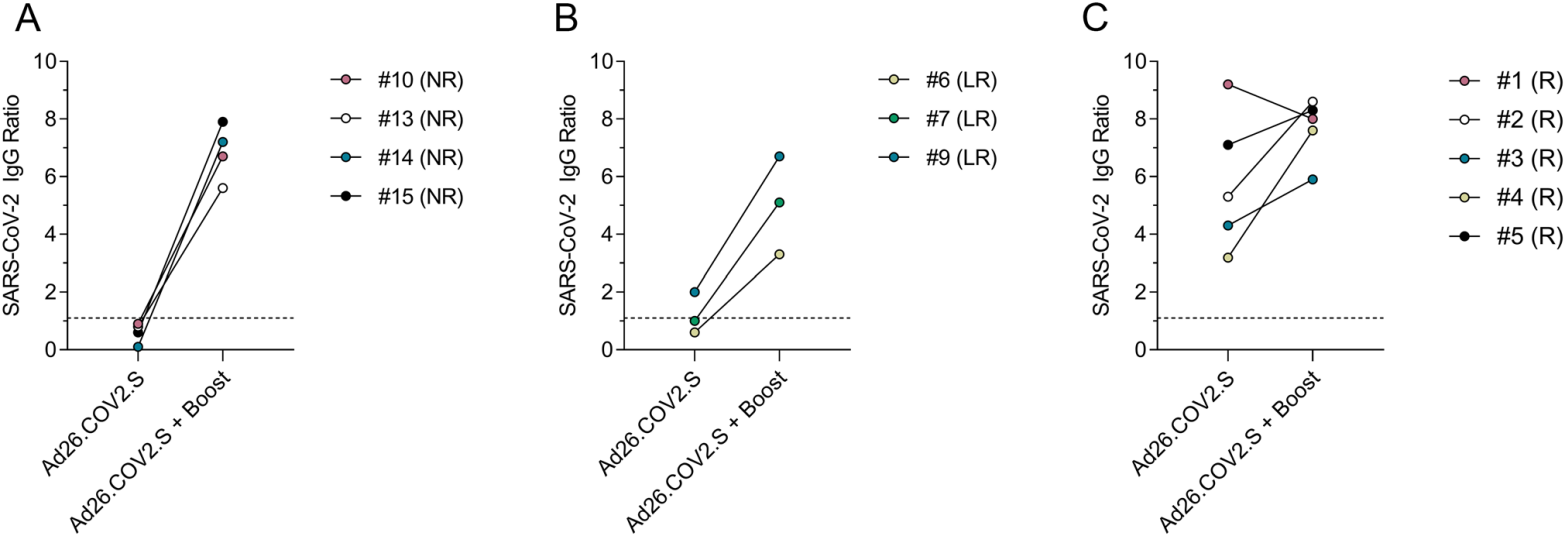
Spike-specific IgG levels after Ad26.COV2.S vaccination and after Ad26.COV2.S + booster vaccination. **A** Spike-specific IgG levels in the group of non-responders. #10 and #13: boosted with BNT162b2 mRNA, #14: boosted with mRNA-1273, #15: boosted with NVX-CoV2373. **B** Spike-specific IgG levels in the group of low responders. All boosted with BNT162b2 mRNA. **C** Spike-specific IgG levels in the group of responders. #1, #2, #3, and #4: boosted with BNT162b2 mRNA, #5: boosted with mRNA-1273. The cutoff for a positive IgG level at 1.1 is indicated by the dashed line

In line with recommendations issued by the German Standing Vaccination Committee [18], four out of six non-responders, three out of five low responders and five out of seven responders were boosted with either an mRNA vaccine (BNT162b2 mRNA, *n* = 9, mRNA-1273, *n* = 2) or a protein subunit vaccine (NVX-CoV2373, *n* = 1). After booster vaccination, all non-responders and low-responders generated SARS-CoV-2 spike-specific antibodies as measured by the Euroimmun ELISA (Fig. 3, supplementary Table S2). The median spike-specific IgG levels in the group of non-responders rose from 0.7 to 7.0 after the booster vaccination. In the group of low responders, the spike-specific IgG levels increased from 1.0 to 5.1 and in the responder group from 5.3 to 8.0. After booster vaccination, the spike-specific IgG levels were not significantly different between Ad26.COV2.S non-responders, low responders and responders, suggesting that the poor antibody response to the Ad26.COV2.S vaccine in our study participants likely reflects a limitation of the vaccine in the context of Ad26 pre-existing immunity rather than a consequence of HIV-1 infection.

## Discussion

In this study, we observed a poor response to Ad26.COV2.S vaccination in HIV-1-infected subjects on antiretroviral therapy. 40% of subjects failed to develop SARS-CoV-2-spike specific antibodies and a further 26% of the vaccinees mounted only low spike antibody titers after vaccination. This poor vaccine response was associated with pre-existing Ad26 neutralizing activity in pre-vaccination sera in a subgroup of patients. This finding contrasts with the results of Ad26.COV2.S phase-1-2a trial in healthy adults [3]. In that trial, a single Ad26.COV2.S vaccination with the same vaccine dose as in our cohort induced SARS-CoV-2-spike specific antibodies in > 96% of the participants in the various trial cohorts [3].

HIV-1-induced immunodeficiency could be a potential factor for a lower vaccine efficacy as it has been demonstrated in cART-treated HIV-1-infected patients for vaccination against hepatitis B [19]. Also in the phase 3 Ensemble trial, vaccine efficacy against moderate to severe Covid-19 at least 14 days after a single Ad26.COV2.S dose was lower in HIV-1-infected vaccinees (vaccine efficacy 15%) than in HIV-1-uninfected individuals (vaccine efficacy 56.9%), but data of the humoral immune response of the HIV-1-infected and HIV-1-uninfected subgroups were not presented in that publication [13].

Nevertheless, we postulate that HIV-1 infection cannot fully explain the observed poor vaccine response in our study. With a median CD4 count of 677 µl (range 336–1177) at the post-vaccination time point and of 619/µl (range 223–1240) at the pre-vaccination time point the low- and non-responders were not severely immunocompromised. Only one non-responder displayed a high viral load of 26 000 copies/ml at the post-vaccination evaluation time point due to non-compliance to antiretroviral therapy, while all other low- and non-responders showed a viral load of ≤ 50 copies/ml. Furthermore, there were no significant differences regarding CD4 counts and viral loads between responders and low-responders or non-responders. In addition, in another group of 50 HIV-1-infected subjects of the Erlangen HIV-cohort, all patients developed spike-specific IgG-antibodies after BNT162b2 mRNA vaccination with titers that were comparable to healthy controls [9]. In that study, CD4 counts and viral loads of the HIV-1-infected subjects were not significantly different from the subjects of this Ad26.COV2.S vaccinated cohort, arguing against HIV-1 infection being a major factor for the observed poor vaccine response. In fact, after heterologous booster vaccination with mRNA- or protein-based vaccines all non-responders mounted antibody responses towards the SARS-CoV-2 spike protein that were not significantly different from the group of Ad26.COV2.S responders. All HIV-1-infected patients had normal or slightly elevated gammaglobulin serum levels ruling out hypogammaglobulinemia as a cause of the low vaccine response rate.

In HIV-1 vaccine trials using adenovirus 5 (Ad5) based vaccines, pre-existing nAbs against adenovirus 5 had a negative effect on the vaccine-induced immune response both in HIV-1-uninfected and HIV-1-infected subjects [20, 21]. In a phase-2-study evaluating an Ad5-based SARS-CoV-2 vaccine, vaccination-induced SARS-CoV-2-RBD-specific antibody levels were approximately two-times lower in vaccinees with high pre-existing anti-Ad5 antibody titers than in participants with low pre-existing anti-Ad5 immunity [22]. The negative impact of pre-existing immunity against Ad5 was a major reason for the development of Ad26-based vectors as the prevalence of antibodies against Ad26 is lower than the prevalence of anti-Ad5 antibodies [23]. While Ad26-seroprevalences was reported to be below 12% in the USA and Europe [23–25], a higher prevalence of Ad26-specific antibodies has been reported with seropositivity rates of 43.1% to 67.8% in various African countries, 54.6% in Thailand [23] and up to 47% in China [26]. Therefore, our findings on the impact of pre-existing Ad26 immunity on Ad26.COV2.S vaccination might be even more relevant in settings with a higher prevalence of Ad26-specific antibodies.

Here, we detected pre-existing serum neutralizing activity against Ad26 in four of six vaccine non-responders and in one of five vaccine low responders, but in none of the seven responders. This neutralizing serum activity affected the expression of both the spike protein and the adenoviral protein. Except for one post-vaccination serum, there was no cross-neutralization of the ChAdOx1-S vector arguing against an unspecific serum effect on protein expression in the HEK293T cells.

In contrast to our findings, another group recently reported that the presence of Ad26- nAbs did not significantly influence the vaccine efficacy of Ad26.COV2.S in healthy subjects in the Ensemble trial [24]. In that trial, 27 of 86 (31%) vaccinees in Brazil, 53 of 80 (66%) vaccinees in South Africa and 1 of 48 (2%) vaccinees in the USA displayed neutralizing activity against Ad26 in their pre-vaccination sera. However, while there were no significant differences regarding the seroconversion rates between subjects with or without pre-existing neutralizing Ad26 antibodies in the South African participants of the Ensemble trial, SARS-CoV-2 spike antibody seroconversion rate and spike antibody titers were significantly lower in Brazilian vaccinees with neutralizing Ad26 antibodies (85% seroconversion, geometric mean concentration (GMC) of spike antibodies: 200) compared to vaccinees without Ad26 nAbs (100% seroconversion, GMC of spike antibodies: 554). The reasons for the discrepant results between the Brazilian and South African cohorts had not been delineated in that study.

Differences in the methodology of measuring Ad26 neutralizing serum activity could contribute to the contrasting results of the Ensemble trial [24] and our study. In the Ensemble trial, Ad26-specific neutralization activity was assessed by serum-mediated inhibition of luciferase expression following transduction of A549 human lung carcinoma cells with an Ad26 construct. In contrast, our neutralization assay used the actual vaccine construct Ad26.COV2.S for transduction of HEK293T cells. As we quantified not only the expression of the adenoviral protein but also the vaccine-induced expression of the spike protein, we directly measured the functional consequences of the presence of Ad26 nAbs. In contrast to the neutralization assay performed in the Ensemble trial [24], we did not perform heat-inactivation of serum as heat inactivation at 56 °C for 30 min may reduce the detection sensitivity of specific antibodies due to protein denaturation and antibody aggregation [27]. It is highly unlikely that the lack of inactivation of complement or HIV-1 had a negative impact on our results by inducing unspecific effects on the cell line as no neutralizing serum activity could be detected in the pre-vaccination sera of the vaccine responders. Furthermore, we did not observe neutralizing activity towards the ChAdOx1 vector in any pre-vaccination sera.

Randomized trials for direct comparison of immunogenicity between mRNA-based SARS-CoV-2 vaccines and Ad26.COV2.S are lacking. However, several cohort studies reported on the induction of lower titers of spike-IgG and -nAbs by the approved single dose of Ad26.COV2.S vaccine in comparison to two-dose vaccination regimens with the mRNA vaccines BNT162b2 mRNA and mRNA-1273 [28–32]. All but one subject in our study developed Ad26-nAbs after vaccination, suggesting that these antibodies might decrease the efficacy of a subsequent Ad26.COV2.S booster. However, in other studies, boosting of Ad26.COV2.S vaccinated subjects with a second dose of the same vaccine was able to increase antibody titers and vaccine efficacy demonstrating that homologous booster vaccination can increase antibody responses in persons likely to have developed Ad26-nAbs after the first dose [31, 33]. Nevertheless, heterologous mRNA-based vaccination boosters induced significantly higher titers of spike antibodies and nAbs in comparison with a homologous Ad26.COV2.S booster [29, 31] indicating a negative impact of pre-existing Ad26-immunity on the Ad26.COV2.S induced SARS-CoV-2-specific humoral immune response.

An important limitation of our study is the retrospective analysis and the limited number of HIV-1-infected subjects. As Ad26.COV2.S became available later than other vaccines, most HIV-1-infected subjects of our cohort had already been vaccinated with mRNA vaccines or the ChAdOx1-S vaccine as they had been prioritized for SARS-CoV-2 vaccination. Recruitment of additional patients vaccinated with only Ad26.COV2.S and available pre-vaccination sera was not possible due to the changes in the recommendations of the German Standing Vaccination Committee [18] limiting the use of Ad26.COV.S vaccine to subjects > 60 years as well as administration of a non-Ad26.COV.2 booster vaccine to all Ad26.COV.2 vaccinees. Nevertheless, the a clear observation of pre-vaccine Ad26-neutralizing antibodies specifically in persons who do not mount antibody responses after Ad26.COV.2 vaccination raises the important point that pre-existing Ad26 antibodies may impede SARS-CoV-2 immunity induced by Ad26.COV.2.

In summary, our findings indicate that pre-existing immunity against Ad26 could be an important contributor to the poor Ad26.COV2.S vaccine response in our HIV cohort. So far, it is not yet known whether these findings also apply to HIV-1-uninfected subjects. Further studies in prospective cohorts of both HIV-1-infected and HIV-1-uninfected vaccinees are needed to define the influence of pre-existing vector immunity on the immunogenicity and vaccine efficacy of the Ad26.COV2.S vaccine.

## Data availability statement

All individual participant data that underlie the results are reported in this article (supplementary Table S1).

### Supplementary Information

Below is the link to the electronic supplementary material.

Supplementary file (PDF 1017 KB)
