# Supplementary material for: Detection of pre-existing neutralizing antibodies against Ad26 in HIV-1-infected individuals not responding to the Ad26.COV2.S vaccine
Source: Infection. 2023 Apr 17;51(6):1657–67. doi: 10.1007/s15010-023-02035-6 (PMC10106868; doi:10.1007/s15010-023-02035-6)
Supplement: Supplementary file 1 — Supplementary file (PDF 1017 KB) [file 15010_2023_2035_MOESM1_ESM.pdf]

Supplementary Material

Detection of pre-existing neutralizing Antibodies against Ad26 in HIV-1-infected Individuals not responding to the Ad26.COV2.S Vaccine

Katja G. Schmidt, Ellen G. Harrer, Verena Schönau, David Simon, Arnd Kleyer, Philipp Steininger, Klaus Korn, Georg Schett, Carina S. Knobloch, Krystelle Nganou-Makamdop, Thomas Harrer*

* Correspondence: Thomas Harrer: Thomas.harrer@uk-erlangen.de

Journal: Infection

Supplementary Figures and Tables

Supplementary Figure S1: FACS plots: Gating strategy and an example of flow cytometric analyses

Supplementary Figure S2: Spike and adenoviral expression as induced by transduction with the Ad26.COV2.S vaccine after addition of pre- and post-vaccination sera

Supplementary Figure S3: Spike and adenoviral expression as induced by transduction with the ChAdOx1-S vaccine after addition of pre- and post-vaccination sera

Supplementary Table S1: Characteristics of study subjects

Supplementary Table S2: Characteristics of study subjects after booster vaccination.

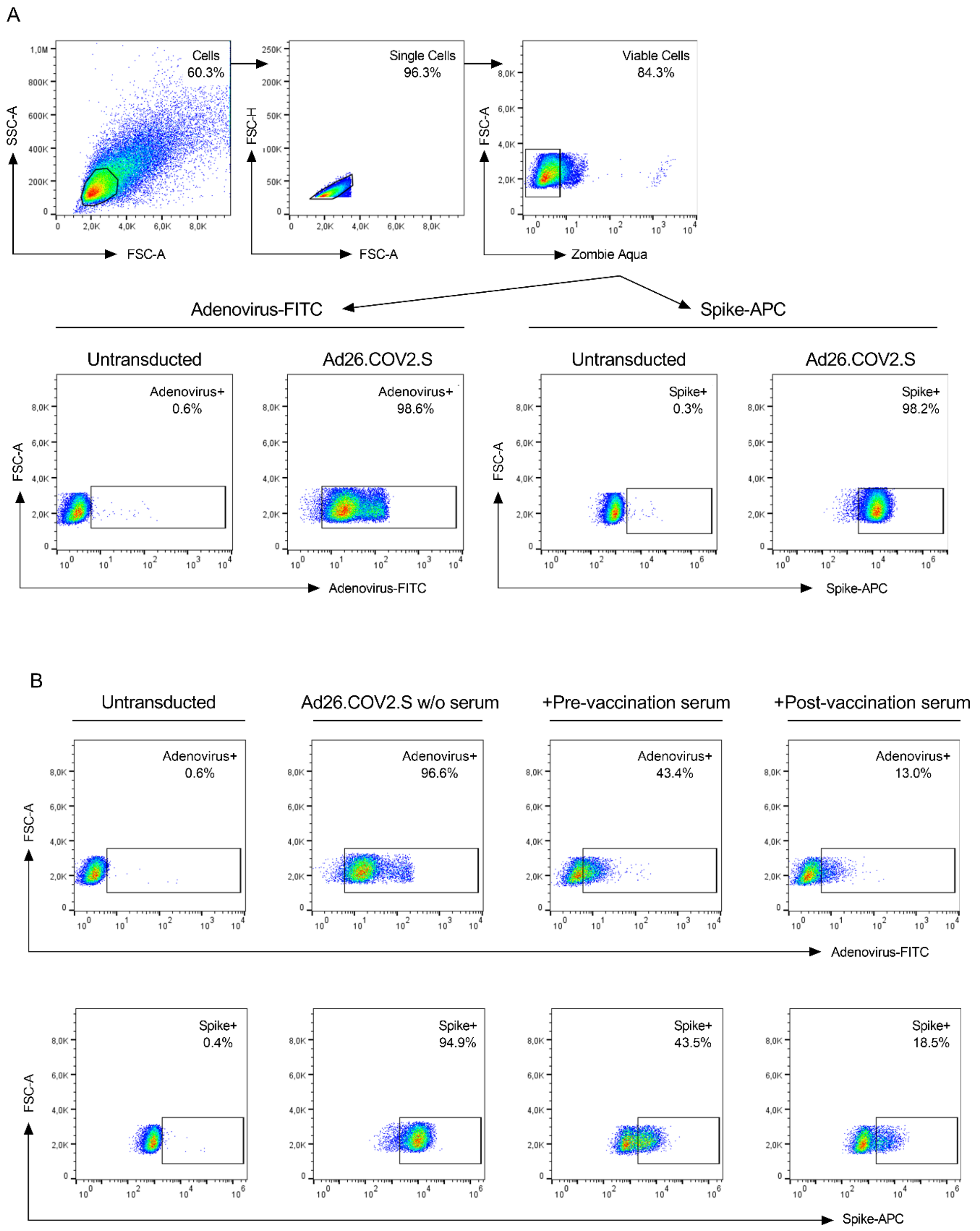

Supplementary Fig. S1 FACS plots. A: Gating Strategy. After gating of cells and single cells, a gate was set on viable cells. Within the viable cells, the adenoviral and spike expression was assessed based on the untransducted control. B: Exemplary plots of blocking effect of pre- and post-vaccination serum of a non-responder (#15).

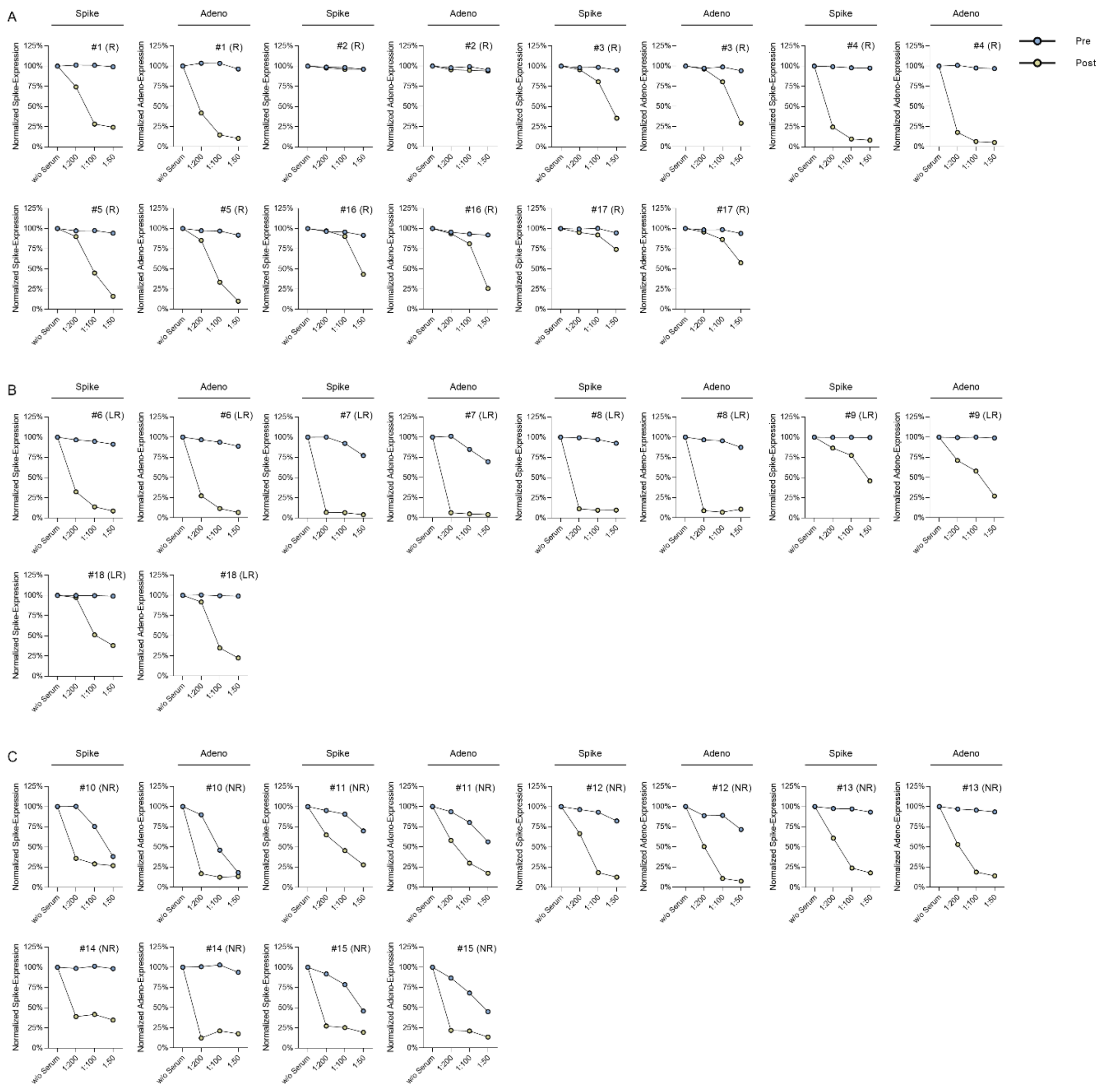

Supplementary Fig. S2 Spike and adenoviral expression as induced by transduction with the Ad26.COV2.S vaccine after addition of pre- and post-vaccination sera. A: Plots of vaccine responders. B: Plots of vaccine low responders. C: Plots of vaccine non-responders.

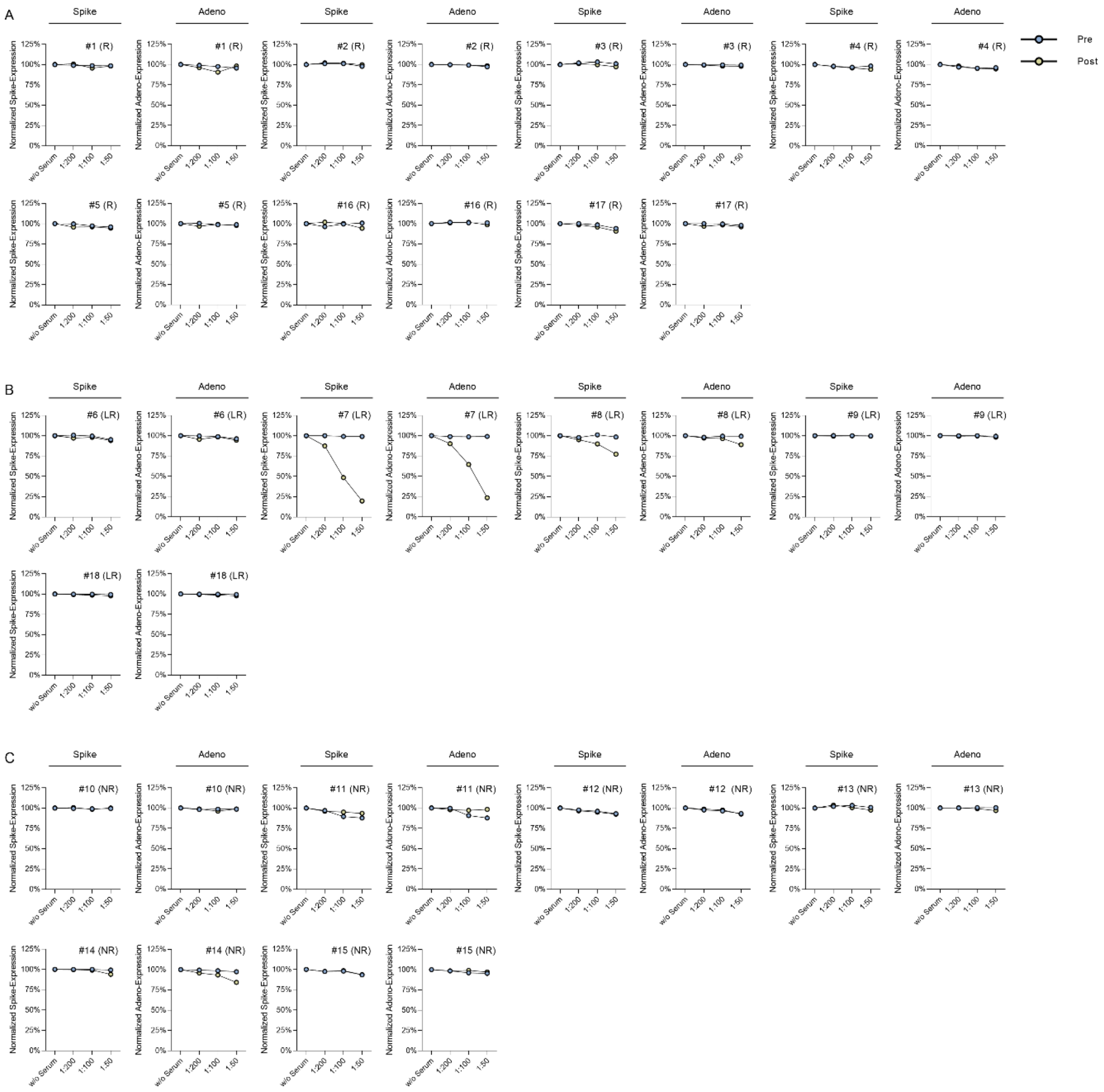

Supplementary Fig. S3 Spike and adenoviral expression as induced by transduction with the ChAdOx1-S vaccine after addition of pre- and post-vaccination sera. A: Plots of vaccine responders. B: Plots of vaccine low responders. C: Plots of vaccine non-responders.

Supplementary Table S1 Characteristics of study subjects

ID	Response	Sex	HIV	Days to Vaccination	Age	Spike ab (BAU/ml)	Spike ab (Ratio)	Gamma-globulins (g/l)	Viral load (copies/ml)	CD4 (cells/µl)	CD8 (cells/µl)	Therapy	Years under therapy
				101[a]	39	10.5	0.2	10.3	<20	780	571	FTC/TAF/COB/DRV	5.8
#1	R	M	Y	0[b]	39	N/A	N/A	N/A	N/A	N/A	N/A	FTC/TAF/COB/DRV	6.1
				21[c]	39	1890	9.2	9.5	<20	768	506	FTC/TAF/COB/DRV	6.1
				150	51	<4.81	0.3	9.5	20	345	383	FTC/TAF/RPV	9.0
#2	R	M	Y	0	52	N/A	N/A	N/A	N/A	N/A	N/A	FTC/TAF/RPV	9.3
				110	52	329	5.3	9.1	<20	281	314	FTC/TAF/RPV	9.6
				890	35	<4.81	0.4	15.5	2500	542	450	/	/
#3	R	F	Y	0	38	N/A	N/A	N/A	N/A	N/A	N/A	DTG, FTC/TDF	2.3
				72	38	318	4.3	11.3	<20	1130	605	DTG, FTC/TDF	2.5
				441	41	<4.81	0.2	8.8	<20	1093	478	FTC/TAF/BIC	12.7
#4	R	M	Y	0	41	N/A	N/A	N/A	N/A	N/A	N/A	FTC/TAF/BIC	13.9
				75	41	148	3.2	9.0	20	1021	429	FTC/TAF/BIC	14.1
				383	44	7.87	0.4	15.4	<20	747	486	FTC/TAF/BIC	14.5
#5	R	F	Y	0	45	N/A	N/A	N/A	N/A	N/A	N/A	FTC/TAF/BIC	15.5
				72	45	519	7.1	14.5	<20	657	465	FTC/TAF/BIC	15.7
				1079	25	<4.81	1.0	13.2	280000	574	1775	/	/
#6	LR	M	Y	0	28	N/A	N/A	N/A	N/A	N/A	N/A	FTC/TDF/DTG	3.0
				101	29	51.5	0.6	9.3	20	1059	1501	FTC/TDF/DTG	3.3
				512	53	16.3	0.2	11.5	<20	657	689	ABC/3TC/DTG	13.6
#7	LR	M	Y	0	54	N/A	N/A	N/A	N/A	N/A	N/A	ABC/3TC/DTG	15.0
				64	54	93.1	1.0	11.7	<20	652	621	ABC/3TC/DTG	15.2
				553	41	<4.81	0.2	19.2	150	686	652	ABC/3TC, DTG	4.5
#8	LR	M	Y	0	43	N/A	N/A	N/A	N/A	N/A	N/A	ABC/3TC, DTG	6.0
				26	43	73.7	0.9	17.0	<20	742	590	ABC/3TC, DTG	6.1
				2426	57	<4.81	0.5	12.8	81	686	1161	RPV, 3TC, TDF	8.5
#9	LR	M	Y	0	63	N/A	N/A	N/A	N/A	N/A	N/A	DTG/3TC, TDF	15.1
				69	64	76.9	2.0	10.8	50	533	809	DTG/3TC, TDF	15.3
				104	64	10.3	0.2	9.5	<20	223	285	FTC/TAF/BIC	3.5
#10	NR	M	Y	0	64	N/A	N/A	N/A	N/A	N/A	N/A	FTC/TAF/BIC	3.8
				64	64	<4.81	0.9	9.6	<20	336	459	FTC/TAF/BIC	4.0
				105	43	<4.81	0.3	11.3	<20	1240	1440	DRV, RTV, MVC, DTG	15.2
#11	NR	M	Y	0	43	N/A	N/A	N/A	N/A	N/A	N/A	DRV, RTV, MVC, RAL	15.5
				21	43	<4.81	0.2	11.6	20	963	1238	DRV, RTV, MVC, RAL	15.5
				16	55	16.2	0.2	11.4	<20	1113	492	FTC/TDF, EFV	12.4
#12	NR	M	Y	0	55	N/A	N/A	N/A	N/A	N/A	N/A	FTC/TDF, EFV	12.5
				109	55	<4.81	0.3	9.8	<20	1177	555	FTC/TDF, EFV	12.8
				116	70	<4.81	0.1	13.3	<20	411	1049	FTC/TAF/BIC	17.8
#13	NR	M	Y	0	70	N/A	N/A	N/A	N/A	N/A	N/A	FTC/TAF/BIC	18.1
				117	71	12.5	0.8	16.1	<20	515	1186	FTC/TAF/BIC	18.4
				83	35	<4.81	0.2	11.0	<20	581	506	ABC/3TC/DTG	3.6
#14	NR	M	Y	0	35	N/A	N/A	N/A	N/A	N/A	N/A	ABC/3TC/DTG	3.8
				11	35	8.42	0.1	11.6	<20	702	612	ABC/3TC/DTG	3.9
				912	43	7.93	0.2	22.0	14000	539	1057	FTC/TAF/DTG	2.5
#15	NR	F	Y	0	46	N/A	N/A	N/A	N/A	N/A	N/A	/	/
				129	46	10.4	0.6	22.0	26000	393	991	/	/
				156	38	57.4	1.3	N/A	/	N/A	N/A	/	/
#16	R	M	N	0	39	N/A	N/A	N/A	/	N/A	N/A	/	/
				151	39	1150	9.1	N/A	/	N/A	N/A	/	/
				173	43	213	4.5	N/A	/	N/A	N/A	/	/
#17	R	M	N	0	43	N/A	N/A	N/A	/	N/A	N/A	/	/
				135	43	1850	11.4	N/A	/	N/A	N/A	/	/
				282	51	<4.81	0.2	N/A	/	N/A	N/A	/	/
#18	LR	M	N	0	51	N/A	N/A	N/A	/	N/A	N/A	/	/
				25	51	34.3	1.1	N/A	/	N/A	N/A	/	/

Abbreviations: M: male, F: female, N: No, Y: Yes, N/A: not available. Response: SARS-CoV-2 spike antibodies after vaccination with Ad26.COV.2. R: responder: >100 BAU/ml, low responders: 33.8 - 100 BAU/ml, non-responders: <33.8 BAU/ml. Spike ab: SARS-CoV-2 spike IgG levels measured by LIAISON SARS-CoV-2 TrimericS IgG assay (BAU/ml) or by Euroimmun (Ratio). Cutoff for a positive antibody titer: ≥33.8 BAU/ml or a ratio of 1.1. The HIV-1-uninfected subjects #16 and #17 had positive SARS-CoV-2 antibodies at their pre-vaccination sample due to prior SARS-CoV-2 infection. Days to vaccination: [a]: days prior to vaccination. [b]: 0 = day of vaccination. [c]: days after vaccination.

Supplementary Table S2. Characteristics of study subjects after booster vaccination.

ID	Response	HIV	Days after boost	Age	Titer (Ratio)	Gamma-globulins (g/l)	Viral load (copies/ml)	CD4 (cells/µl)	CD8 (cells/µl)	Therapy	Years under therapy
#1	R	Y	47	40	8.0	9·7	<20	859	486	FTC/TAF/COB/DRV	6.6
#2	R	Y	36	52	8.6	11·0	<20	325	388	FTC/TAF/BIC	9.9
#3	R	Y	25	38	5.9	11·1	<20	1503	847	DTG, FTC/TDF	2.8
#4	R	Y	106	42	7.6	8·9	<20	1158	509	FTC/TAF/BIC	14.8
#5	R	Y	80	46	8.3	15·1	<20	651	434	FTC/TAF/BIC	16.4
#6	LR	Y	125	29	3.3	9·4	20	1499	2026	FTC/TDF/DTG	3.6
#7	LR	Y	118	54	5.1	11·8	<20	684	717	ABC/3TC/DTG	15.5
#9	LR	Y	82	64	6.7	11·5	60	550	632	DTG/3TC, TDF	15.9
#10	NR	Y	55	65	6.7	9.3	<20	366	537	FTC/TAF/BIC	4.5
#13	NR	Y	32	71	5.6	14.0	<20	343	715	FTC/TAF/BIC	18.6
#14	NR	Y	84	36	7.2	11.3	<20	656	544	ABC/3TC/DTG	4.6
#15	NR	Y	141	47	7.9	18.1	14000	371	758	/	/

Abbreviations: Response: Response to Ad26.COV2.S vaccine. R: responder, LR: low responder, NR: non-responder; N: No, Y: Yes; Titer: SARS-CoV-2 spike IgG levels measured by Euroimmun (Ratio). Cutoff for a positive antibody titer: ratio of ≥ 1.1.
